# Complete genome sequence of *Marivirga tractuosa* type strain (H-43^T^)

**DOI:** 10.4056/sigs.1623941

**Published:** 2011-04-29

**Authors:** Ioanna Pagani, Olga Chertkov, Alla Lapidus, Susan Lucas, Tijana Glavina Del Rio, Hope Tice, Alex Copeland, Jan-Fang Cheng, Matt Nolan, Elizabeth Saunders, Sam Pitluck, Brittany Held, Lynne Goodwin, Konstantinos Liolios, Galina Ovchinikova, Natalia Ivanova, Konstantinos Mavromatis, Amrita Pati, Amy Chen, Krishna Palaniappan, Miriam Land, Loren Hauser, Cynthia D. Jeffries, John C. Detter, Cliff Han, Roxanne Tapia, Olivier D. Ngatchou-Djao, Manfred Rohde, Markus Göker, Stefan Spring, Johannes Sikorski, Tanja Woyke, Jim Bristow, Jonathan A. Eisen, Victor Markowitz, Philip Hugenholtz, Hans-Peter Klenk, Nikos C. Kyrpides

**Affiliations:** 1 DOE Joint Genome Institute, Walnut Creek, California, USA; 2 Los Alamos National Laboratory, Bioscience Division, Los Alamos, New Mexico, USA; 3 Biological Data Management and Technology Center, Lawrence Berkeley National Laboratory, Berkeley, California, USA; 4 Oak Ridge National Laboratory, Oak Ridge, Tennessee, USA; 5 HZI – Helmholtz Centre for Infection Research, Braunschweig, Germany; 6 DSMZ – German Collection of Microorganisms and Cell Cultures GmbH, Braunschweig, Germany; 7 University of California Davis Genome Center, Davis, California, USA; 8 Australian Centre for Ecogenomics, School of Chemistry and Molecular Biosciences, The University of Queensland, Brisbane, Australia

**Keywords:** mesophilic, chemoorganotrophic, strictly aerobic, Gram-negative, slender and flexible rod-shaped, non-sporeforming, motile by gliding, *Flammeovirgaceae*, GEBA

## Abstract

*Marivirga tractuosa* (Lewin 1969) Nedashkovskaya *et al*. 2010 is the type species of the genus *Marivirga*, which belongs to the family *Flammeovirgaceae*. Members of this genus are of interest because of their gliding motility. The species is of interest because representative strains show resistance to several antibiotics, including gentamicin, kanamycin, neomycin, polymixin and streptomycin. This is the first complete genome sequence of a member of the family *Flammeovirgaceae*. Here we describe the features of this organism, together with the complete genome sequence and annotation. The 4,511,574 bp long chromosome and the 4,916 bp plasmid with their 3,808 protein-coding and 49 RNA genes are a part of the *** G****enomic* *** E****ncyclopedia of* *** B****acteria and* *** A****rchaea * project.

## Introduction

Strain H-43^T^ (= DSM 4126 = ATCC 23168 = NBRC 15989) is the type strain of the species *Marivirga tractuosa.* The genus *Marivirga*, whose type species is *M. tractuosa*, contains only one additional species: *M. sericea*. The generic name ‘Marivirga’ derives from Latin words ‘mare’, the sea and ‘virga’, rod, meaning ‘a rod that inhabits marine environments’ [1]. The species epithet ‘tractuosa’ is a Latin adjective meaning ‘that draws to itself, gluey, viscous’, probably referring to the phenotype of gliding motility [1]. Strain H-43^T^ was isolated in 1969 from a beach sand sample collected from Nhatrang (South China Sea), Vietnam [2] and was initially named ‘*Microscilla tractuosa*’ by Lewin [3], but was never validly published under this name. The strain was then in 1974 joined to the genus *Flexibacter* by Leadbetter [4]. In 2010, strain H-43^T^ was reclassified to the novel genus *Marivirga,* based on a polyphasic approach [1]. Other strains have been isolated worldwide from mud in the Orne Estuary, France and silty sand in Penang, Malaysia [5], as well as from brown mud from Muigh Inis, Ireland, underneath frozen sand in the upper littoral zone at Auke Bay, Alaska, red-brown mud from Helgoland Island, Germany, and from brown sand at Moreton Bay, Australia [6]. These sampling sites suggest an ecological preference of *M. tractuosa* for wet terrestrial habitats [1,2]. Here we present a summary classification and a set of features for *M. tractuosa* strain H-43^T^, together with the description of the complete genomic sequencing and annotation.

## Classification and features

**Figure 1:**
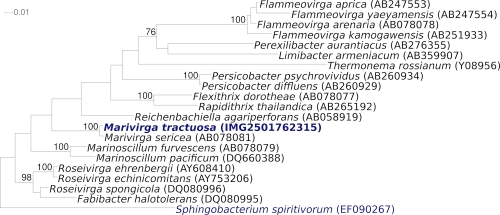
Phylogenetic tree highlighting the position of *M. tractuosa* relative to the other type strains within the family *Flammeovirgaceae*. The trees were inferred from 1,408 aligned characters [7,8] of the 16S rRNA gene sequence under the maximum likelihood criterion [9] and rooted in accordance with the family *Sphingobacteriaceae*. The branches are scaled in terms of the expected number of substitutions per site. Numbers above branches are support values from 1,000 bootstrap replicates [10] if larger than 60%. Lineages with type strain genome sequencing projects registered in GOLD [11] are shown in blue, published genomes in bold.

The 16S rRNA gene sequence of the strain H-43^T^ shares the highest degree of similarity (99.1%) with *M. sericea*, the only other member of the genus *Marivirga* (Figure 1) [12], and with an uncultured *Bacteroidetes* clone SHBC423 (99%, GQ350249) from oceanic dead zones [13]. A representative genomic 16S rRNA gene sequence of *M. tractuosa* was compared using NCBI BLAST under default values with the most recent release of the Greengenes database [14] and the relative frequencies, weighted by BLAST scores, of taxa and keywords (reduced to their stem [15]) were determined. The five most frequent genera were *Flexibacter* (= not yet renamed *Marivirga* hits) (26.8%), *Pontibacter* (21.6%), *Hymenobacter* (21.4%), *Adhaeribacter* (8.3%) and *Microscilla* (8.0%) (57 hits in total). The highest-scoring environmental sequence was EU447282 ('*Flexibacteraceae* bacterium KMM 6276'), which showed an identity of 100.0% and an HSP coverage of 97.6%, but most probably represents a *Marivirga* strain. The five most frequent keywords within the labels of environmental samples which yielded hits were 'microbi' (4.0%), 'sediment' (3.1%), 'site' (1.9%), 'group' (1.7%) and 'coral' (1.6%) (192 hits in total). These keywords support the ecological preference of *M. tractuosa* for wet habitats, as deduced from the sampling sites of the cultivated strains. Environmental samples which yielded hits of a higher score than the highest scoring species were not found.

Figure 1 shows the phylogenetic neighborhood of *M. tractuosa* H-43^T^ in a 16S rRNA based tree. The sequences of the two identical 16S rRNA gene copies in the genome do not differ from the previously published 16S rRNA sequence (AB078072).

**Figure 2:**
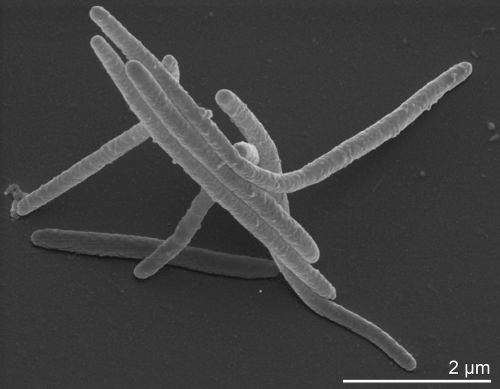
Scanning electron micrograph of *M. tractuosa* H-43^T^

The cells of strain H-43^T^ are long, slender and flexible rods 0.4-0.5 µm in diameter and 10-50 µm in length or longer (Figure 2). Strain H-43^T^ is a Gram-negative non-spore-forming bacterium (Table 1) that exhibits gliding motility [1]. Strain H-43^T^ is strictly aerobic  and chemoorganotrophic [1]. Growth is observed at 10-40ºC and with 0.5–10% NaCl, with optimal growth at 28-32ºC and 4-7% NaCl [1]. Colonies are circular, shiny and 2-4 mm in diameter after 72 h of incubation on marine agar [1]. They are usually dark-orange in color but whitish or yellow-pigmented variants may occur [1]. Pigment type three was found in the strain H-43^T^, the main pigment being saproxanthin [2]. In *n*-hexane, the absorption maxima of the pigments from crude extract were 425 nm, 447 nm, 471 nm and 505 nm [2]. Flexirubin-type pigments are not produced. Arginine dihydrolase, ornithine decarboxylase, lysine decarboxylase and tryptophan deaminase activities were described to be absent [1], however, Srinivas *et al*. [22] found that strain H-43^T^ could utilize arginine, and also that growth on alanine and cysteine was weak. Nitrate is not reduced. Indole and acetoin (Voges–Proskauer reaction) are not produced [1]. Gelatin, Tween 20, Tween 40, Tween 80 and DNA are hydrolyzed, as well as agar, starch, urea, cellulose (CM-cellulose and filter paper) and chitin [1,2], however, again in contrast to the original description [1], Srinivas *et al*. reported that the strain does not hydrolyze Tween 20, Tween 40 or Tween 80 [22]. Acid is not produced from L-arabinose, cellobiose, L-fucose, D-galactose, glycerol, lactose, melibiose, raffinose, L-rhamnose, L-sorbose, sucrose, trehalose, DL-xylose, N-acetylglucosamine, citrate, acetate, fumarate, malate, adonitol, dulcitol, inositol or mannitol. In the API 50 CH gallery, acid is produced only from esculin and arbutin. Production of hydrogen sulfide and hydrolysis of casein are variable [1]. Citrate is utilized but lactose, inositol, gluconate, caprate, phenylalanine and malonate are not. Utilization of arabinose, D-glucose, D-mannose, sucrose, mannitol, N-acetylglucosamine, maltose, adipate, malate and sorbitol is variable [1]. Glucose, glycerol, galactose and sucrose (5.1 g/l, each) are used as carbon sources and stimulate the growth of strain H-43^T^, while sodium acetate and sodium lactate do not [2]. Nitrogen sources supporting growth include tryptone (1 g/l) and casamino acids (1 g/l), but not sodium glutamate or NO_3_^-^ [2]. Alkaline phosphatase, esterase (C4), esterase lipase (C8), leucine arylamidase, valine arylamidase, cystine arylamidase, α-chymotrypsin, acid phosphatase, naphthol-AS-BI-phosphohydrolase, β-galactosidase and α- and β-glucosidase activities are present, but lipase (C14), trypsin, α-galactosidase, β-glucuronidase, N-acetyl β-glucosaminidase, α-mannosidase and α-fucosidase activities are negative in the API ZYM gallery [1]. In litmus-milk, the dye was reduced and the clotting occurred. Moreover, litmus turned pink due to acidification and the curd was re-digested because of proteolysis [2]. Strain H-43^T^ is sensitive to ampicillin (10 µg), benzylpenicillin (10 U), carbenicillin (100 µg), chloramphenicol (30 µg), doxycycline (10 µg), erythromycin (15 µg), lincomycin (15 µg), oleandomycin (15 µg) and tetracycline (30 µg), but resistant to gentamicin (10 µg), kanamycin (30 µg), neomycin (30 µg), polymixin (300 U) and streptomycin (30 µg) [1]. Cytochrome oxidase, catalase and alkaline phosphatase tests were positive [1], although Srinivas *et al*. [22] found only a weak reaction in the catalase test. When growing, the strain was able to degrade dihydroxyphenyl alanine and tyrosine (5 g/l) [2].

**Table 1:** Classification and general features of *M. tractuosa* H-43^T^ according to the MIGS recommendations [16]

**MIGS ID**	**Property**	**Term**	**Evidence code**
	Current classification	Domain *Bacteria*	TAS [17]
		Phylum *Bacteroidetes*	TAS [19]
		Class *Sphingobacteria*	TAS [18]
		Order *Sphingobacteriales*	TAS [18]
		Family *Flammeovirgaceae*	TAS [18]
		Genus *Marivirga*	TAS [1]
		Species *Marivirga tractuosa*	TAS [1]
		Type strain H-43	TAS [1]
	Gram stain	negative	TAS [1,2]
	Cell shape	long, slender and flexible rods	TAS [1]
	Motility	motile by gliding	TAS [1,2]
	Sporulation	no	TAS [1,2]
	Temperature range	10°C-40°C	TAS [1]
	Optimum temperature	28°C-32°C	TAS [1,2]
	Salinity	0.5%-10% NaCl	TAS [1]
MIGS-22	Oxygen requirement	strictly aerobic	TAS [1,2]
	Carbon source	glycerol, glucose, galactose, sucrose	TAS [2]
	Energy source	chemoorganotroph	TAS [1]
MIGS-6	Habitat	wet terrestrial habitats, occasionally fresh water	TAS [2]
MIGS-15	Biotic relationship	free-living	NAS
MIGS-14	Pathogenicity	not reported	NAS
	Biosafety level	1	TAS [20]
	Isolation	beach sand sample	TAS [1]
MIGS-4	Geographic location	Nhatrang (South China Sea), Vietnam	TAS [1]
MIGS-5	Sample collection time	1969 or before	TAS [2]
MIGS-4.1 MIGS-4.2	Latitude Longitude	12.25 109.20	NAS
MIGS-4.3	Depth	not reported	NAS
MIGS-4.4	Altitude	not reported	NAS

Evidence codes - IDA: Inferred from Direct Assay (first time in publication); TAS: Traceable Author Statement (i.e., a direct report exists in the literature); NAS: Non-traceable Author Statement (i.e., not directly observed for the living, isolated sample, but based on a generally accepted property for the species, or anecdotal evidence). These evidence codes are from of the Gene Ontology project [21]. If the evidence code is IDA, then the property was directly observed by one of the authors or an expert mentioned in the acknowledgements

### Chemotaxonomy

The predominant cellular fatty acid of the strain H-43^T^ were *iso*-C_15:0_ (36.8%), *iso*-C_15:1_ (23.0%) and *iso*-C_17:03-OH_ (12.2%), with a detailed listing given in Nedashkovskaya *et al*. [1]. Srinivas *et al*. reported fundamentally different observations for strain H-43^T^, with the C_16:0_ (69% of the total fatty acids) to be the most important fatty acids in the strain H-43^T^, whereas *iso*-C_15:0_ was not detectable [22]. The main respiratory quinone is MK-7 [1].

## Genome sequencing and annotation

### Genome project history

This organism was selected for sequencing on the basis of its phylogenetic position [23], and is part of the *** G****enomic* *** E****ncyclopedia of* *** B****acteria and* *** A****rchaea * project [24]. The genome project is deposited in the Genomes On Line Database [11] and the complete genome sequence is deposited in GenBank. Sequencing, finishing and annotation were performed by the DOE Joint Genome Institute (JGI). A summary of the project information is shown in Table 2.

**Table 2:** Genome sequencing project information

**MIGS ID**	**Property**	**Term**
MIGS-31	Finishing quality	Finished
MIGS-28	Libraries used	Three genomic libraries: one 454 pyrosequence standard library, one 454 PE library (10 kb insert size), one Illumina library
MIGS-29	Sequencing platforms	Illumina GAii, 454 GS FLX Titanium
MIGS-31.2	Sequencing coverage	60.1 × Illumina; 44.4 × pyrosequence
MIGS-30	Assemblers	Newbler version 2.1-PreRelease-4-28-2009-gcc-3.4.6-threads, Velvet, phrap
MIGS-32	Gene calling method	Prodigal 1.4, GenePRIMP
	INSDC ID	CP002349 (chromosome) CP002350 (plasmid FTRAC01)
	Genbank Date of Release	December 7, 2010
	GOLD ID	Gc01555
	NCBI project ID	37901
	Database: IMG-GEBA	2503538019
MIGS-13	Source material identifier	DSM 4126
	Project relevance	Tree of Life, GEBA

### Growth conditions and DNA isolation

*M. tractuosa* H-43^T^, DSM 4126, was grown in DSMZ medium 172 (Cytophaga (marine) medium) [25] at 25°C. DNA was isolated from 0.5-1 g of cell paste using MasterPure Gram-positive DNA purification kit (Epicentre MGP04100) following the standard protocol as recommended by the manufacturer with modification st/DL for cell lysis as described in Wu *et al*. [24]. DNA is available through the DNA Bank Network [26,27].

### Genome sequencing and assembly

The genome was sequenced using a combination of Illumina and 454 sequencing platforms. All general aspects of library construction and sequencing can be found at the JGI website [28]. Pyrosequencing reads were assembled using the Newbler assembler version 2.1-Pre-release-4-28-2009-gcc-3.4.6-threads (Roche). The initial Newbler assembly consisted of 115 contigs in one scaffold and was converted into a phrap [29] assembly by making fake reads from the consensus, collecting the read pairs in the 454 paired end library. Illumina GAii sequencing data (496 Mb) was assembled with Velvet [30] and the consensus sequences were shredded into 1.5 kb overlapped fake reads and assembled together with the 454 data. The 454 draft assembly was based on 201.9 Mb 454 draft data and all of the 454 paired end data. Newbler parameters are -consed -a 50 -l 350 -g -m -ml 20. The Phred/Phrap/Consed software package [29] was used for sequence assembly and quality assessment in the following finishing process. After the shotgun stage, reads were assembled with parallel phrap (High Performance Software, LLC). Possible mis-assemblies were corrected with gapResolution [28], Dupfinisher, or sequencing cloned bridging PCR fragments with subcloning or transposon bombing (Epicentre Biotechnologies, Madison, WI) [31]. Gaps between contigs were closed by editing in Consed, by PCR and by Bubble PCR primer walks (J.-F.Chang, unpublished). A total of 336 additional reactions were necessary to close gaps and to raise the quality of the finished sequence. Illumina reads were also used to correct potential base errors and increase consensus quality using a software Polisher developed at JGI [32]. The error rate of the completed genome sequence is less than 1 in 100,000. Together, the combination of the Illumina and 454 sequencing platforms provided 104.5 × coverage of the genome. Final assembly contains 589,653 pyrosequence and 7,543,442 Illumina reads.

### Genome annotation

Genes were identified using Prodigal [33] as part of the Oak Ridge National Laboratory genome annotation pipeline, followed by a round of manual curation using the JGI GenePRIMP pipeline [34]. The predicted CDSs were translated and used to search the National Center for Biotechnology Information (NCBI) nonredundant database, UniProt, TIGRFam, Pfam, PRIAM, KEGG, COG, and InterPro databases. Additional gene prediction analysis and functional annotation was performed within the Integrated Microbial Genomes - Expert Review (IMG-ER) platform [35].

## Genome properties

**Figure 3:**
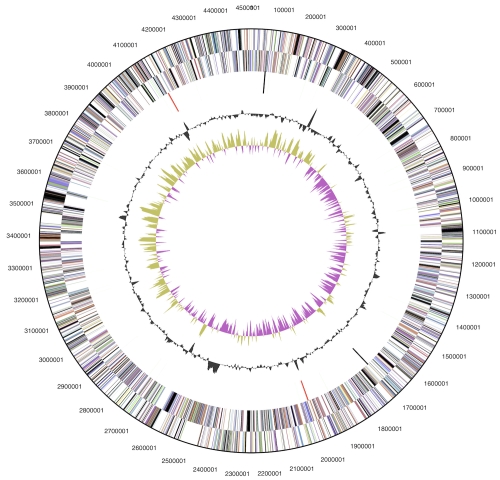
Graphical circular map of the chromosome (plasmid map not shown). From outside to the center: Genes on forward strand (color by COG categories), Genes on reverse strand (color by COG categories), RNA genes (tRNAs green, rRNAs red, other RNAs black), GC content, GC skew.

The genome consists of a 4,511,574 bp long chromosome with a 35.5% G+C content and a 4,916 bp plasmid with 40% G+C content (Table 3 and Figure 3). Of the 3,857 genes predicted, 3,808 were protein-coding genes, and 49 RNAs; Fifty-one pseudogenes were identified. The majority of the protein-coding genes (62.2%) were assigned with a putative function while the remaining ones were annotated as hypothetical proteins. The distribution of genes into COGs functional categories is presented in Table 4.

**Table 3:** Genome Statistics

**Attribute**	**Value**	**% of Total**
Genome size (bp)	4,516,490	100.00%
DNA coding region (bp)	4,029,412	89.22%
DNA G+C content (bp)	1,604,111	35.52%
Number of replicons	2	
Extrachromosomal elements	1	
Total genes	3,857	100.00%
RNA genes	49	1.27%
rRNA operons	2	
Protein-coding genes	3,808	98.73%
Pseudo genes	51	1.32%
Genes with function prediction	2,398	62.17%
Genes in paralog clusters	396	10.27%
Genes assigned to COGs	2,375	61.58%
Genes assigned Pfam domains	2,609	67.64%
Genes with signal peptides	1,113	28.86%
Genes with transmembrane helices	997	25.85%
CRISPR repeats	0	

**Table 4:** Number of genes associated with the general COG functional categories

**Code**	**value**	**% age**	**Description**
J	157	6.1	Translation, ribosomal structure and biogenesis
A	0	0.0	RNA processing and modification
K	163	6.3	Transcription
L	131	5.1	Replication, recombination and repair
B	1	0.1	Chromatin structure and dynamics
D	30	1.2	Cell cycle control, cell division, chromosome partitioning
Y	0	0.0	Nuclear structure
V	63	2.4	Defense mechanisms
T	184	7.1	Signal transduction mechanisms
M	236	9.1	Cell wall/membrane/envelope biogenesis
N	10	0.4	Cell motility
Z	1	0.0	Cytoskeleton
W	0	0.0	Extracellular structures
U	37	1.4	Intracellular trafficking and secretion, and vesicular transport
O	112	4.3	Posttranslational modification, protein turnover, chaperones
C	126	4.9	Energy production and conversion
G	102	3.9	Carbohydrate transport and metabolism
E	217	8.4	Amino acid transport and metabolism
F	67	2.6	Nucleotide transport and metabolism
H	118	4.6	Coenzyme transport and metabolism
I	99	3.8	Lipid transport and metabolism
P	136	5.3	Inorganic ion transport and metabolism
Q	51	2.0	Secondary metabolites biosynthesis, transport and catabolism
R	340	13.1	General function prediction only
S	208	8.0	Function unknown
-	1,482	38.4	Not in COGs

## References

[1] Nedashkovskaya OI Vancanneyt M Kim SB Bae KS Reclassification of *Flexibacter tractuosus* (Lewin 1969) Leadbetter 1974 and '*Microscilla sericea*' Lewin 1969 in the genus *Marivirga* gen. nov. as *Marivirga tractuosa* comb. nov. and *Marivirga sericea* nom. rev., comb. nov. Int J Syst Evol Microbiol 2010; 60:1858-1863 10.1099/ijs.0.016121-0 19767357

[2] Lewin RA Lounsbery DM Isolation, cultivation and characterization of flexibacteria. J Gen Microbiol 1969; 58:145-170 5360473 10.1099/00221287-58-2-145

[3] Lewin RA A classification of flexibacteria. J Gen Microbiol 1969; 58:189-206 5360476 10.1099/00221287-58-2-189

[4] Leadbetter ER. 1974.Genus II. *Flexibacter* Soriano 1945, 92, Lewin 1969, 192 emend. mut. char. *In:* Buchanan RE, Gibbons NE (*eds*), Bergey's Manual of Determinative Bacteriology, Eighth Edition, The Williams and Wilkins Co., Baltimore, p. 105-107.

[5] American Type Culture Collection. http://www.atcc.org

[6] National Collection of Industrial Bacteria http://www.ncimb.com

[7] Castresana J Selection of conserved blocks from multiple alignments for their use in phylogenetic analysis. Mol Biol Evol 2000; 17:540-552 10742046 10.1093/oxfordjournals.molbev.a026334

[8] Lee C Grasso C Sharlow MF Multiple sequence alignment using partial order graphs. Bioinformatics 2002; 18:452-464 10.1093/bioinformatics/18.3.452 11934745

[9] Stamatakis A Hoover P Rougemont J A rapid bootstrap algorithm for the RAxML web servers. Syst Biol 2008; 57:758-771 10.1080/10635150802429642 18853362

[10] Pattengale ND Alipour M Bininda-Emonds ORP Moret BME Stamatakis A How many bootstrap replicates are necessary? Lect Notes Comput Sci 2009; 5541:184-200 10.1007/978-3-642-02008-7_13 20377449

[11] Liolios K Mavromatis K Tavernarakis N Kyrpides NC The Genomes On Line Database (GOLD) in 2007: status of genomic and metagenomic projects and their associated metadata. Nucleic Acids Res 2008; 36:D475-D479 10.1093/nar/gkm884 17981842 PMC2238992

[12] Nakagawa Y Sakane T Suzuki M Hatano K Phylogenetic structure of the genera *Flexibacter, Flexithrix*, and *Microscilla* deduced from 16S rRNA sequence analysis. J Gen Appl Microbiol 2002; 48:155-165 10.2323/jgam.48.155 12469298

[13] Walsh DA Zaikova E Howes CG Song YC Wright JJ Tringe SG Tortell PD Hallam SJ Metagenome of a versatile chemolithoautotroph from expanding oceanic dead zones. Science 2009; 326:578-582 10.1126/science.1175309 19900896

[14] DeSantis TZ Hugenholtz P Larsen N Rojas M Brodie E Keller K Huber T Dalevi D Hu P Andersen G Greengenes, a chimera-checked 16S rRNA gene database and workbench compatible with ARB. Appl Environ Microbiol 2006; 72:5069-5072 10.1128/AEM.03006-05 16820507 PMC1489311

[15] Porter MF An algorithm for suffix stripping. Program: electronic library and information systems 1980; 14:130-137 10.1108/eb046814

[16] Field D Garrity G Gray T Morrison N Selengut J Sterk P Tatusova T Thomson N Allen MJ Angiuoli SV The minimum information about a genome sequence (MIGS) specification. Nat Biotechnol 2008; 26:541-547 10.1038/nbt1360 18464787 PMC2409278

[17] Woese CR Kandler O Wheelis ML Towards a natural system of organisms: proposal for the domains *Archaea, Bacteria*, and *Eucarya.* Proc Natl Acad Sci USA 1990; 87:4576-4579 10.1073/pnas.87.12.4576 2112744 PMC54159

[18] Garrity GM, Holt JG. 2001. Taxonomic Outline of the *Archaea* and *Bacteria* In: Garrity GM, Boone DR, Castenholz RW (eds), Bergey's Manual of Systematic Bacteriology, Second Edition, Volume 1, Springer, New York, p. 155-166.

[19] Garrity GM, Holt JG. The Road Map to the Manual. In: Garrity GM, Boone DR, Castenholz RW (eds), Bergey's Manual of Systematic Bacteriology, Second Edition, Volume 1, Springer, New York, 2001, p. 119-169

[20] Classification of bacteria and archaea in risk groups. http://www.baua.de TRBA 466.

[21] Ashburner M Ball CA Blake JA Botstein D Butler H Cherry JM Davis AP Dolinski K Dwight SS Eppig JT Gene Ontology: tool for the unification of biology. Nat Genet 2000; 25:25-29 10.1038/75556 10802651 PMC3037419

[22] Srinivas TNR Anil Kumar P Madhu S Sunil B Sharma TVRS Shivaji S *Cesiribacter andamanensis* gen. nov., sp. nov., a novel bacterium isolated from a soil sample of a mud volcano, Andaman Islands, India. Int J Syst Evol Microbiol 2010; (In press). 10.1099/ijs.0.025429-0 20656812

[23] Klenk HP Göker M En route to a genome-based classification of *Archaea* and *Bacteria*? Syst Appl Microbiol 2010; 33:175-182 10.1016/j.syapm.2010.03.003 20409658

[24] Wu D Hugenholtz P Mavromatis K Pukall R Dalin E Ivanova NN Kunin V Goodwin L Wu M Tindall BJ A phylogeny-driven genomic encyclopaedia of *Bacteria* and *Archaea*. Nature 2009; 462:1056-1060 10.1038/nature08656 20033048 PMC3073058

[25] List of growth media used at DSMZ: http://www.dsmz.de/microorganisms/media_list.php

[26] Gemeinholzer B Dröge G Zetzsche H Haszprunar G Klenk HP Güntsch A Berendsohn WG Wägele JW The DNA Bank Network: the start from a German initiative. Biopreservation and Biobanking 2011; 9:51-55 10.1089/bio.2010.0029 24850206

[27] DNA bank Network www.dnabank-network.org

[28] DOE Joint Genome Institute http://www.jgi.doe.gov/

[29] Phrap and Phred for Windows. MacOS, Linux, and Unix. http://www.phrap.com

[30] Zerbino DR Birney E Velvet: algorithms for de novo short read assembly using de Bruijn graphs. Genome Res 2008; 18:821-829 10.1101/gr.074492.107 18349386 PMC2336801

[31] Sims D Brettin T Detter J Han C Lapidus A Copeland A Glavina Del Rio T Nolan M Chen F Lucas S Complete genome sequence of *Kytococcus sedentarius* type strain (541^T^). Stand Genomic Sci 2009; 1:12-20 10.4056/sigs.761 21304632 PMC3035214

[32] Lapidus A, LaButti K, Foster B, Lowry S, Trong S, Goltsman E. POLISHER: An effective tool for using ultra short reads in microbial genome assembly and finishing. AGBT, Marco Island, FL, 2008.

[33] Hyatt D Chen GL LoCascio PF Land ML Larimer FW Hauser LJ Prodigal: prokaryotic gene recognition and translation initiation site identification. BMC Bioinformatics 2010; 11:119 10.1186/1471-2105-11-119 20211023 PMC2848648

[34] Pati A Ivanova N Mikhailova N Ovchinikova G Hooper SD Lykidis A Kyrpides NC GenePRIMP: A gene prediction improvement pipeline for microbial genomes. Nat Methods 2010; 7:455-457 10.1038/nmeth.1457 20436475

[35] Markowitz VM Ivanova NN Chen IMA Chu K Kyrpides NC IMG ER: a system for microbial genome annotation expert review and curation. Bioinformatics 2009; 25:2271-2278 10.1093/bioinformatics/btp393 19561336

